# Cold Applications to Aching Teeth

**Published:** 1866-02

**Authors:** 


### COLD APPLICATIONS TO ACHING TEETH.

ÆSOP tells us of a Satyr who kicked a traveler out of his cavern, where he had been kindly entertaining him, because he blew his breath on his fingers to warm them and on his victuals to cool them. Blowing hot and cold with the same breath being a mystery he could not fathom, he regarded it as an inconsistency he would not tolerate within his premises. But the traveler was right in his head, and true to the laws of caloric, nevertheless. The Satyr had, simply, failed to study the philosophy of heat. Many mistakes like that of the Satyr occur in our profession.

Almost every one knows that cold applications sometimes relieve the pain of toothache, and sometimes aggravate it. I once heard an aged and experienced member of our profession say that "this is the greatest mystery of our specialty." The Satyr had neglected the laws of heat; our professional father had not mastered the principles of pathology.

When there is an increase in the quantity of blood in any part or tissue of the body, with increased motion of it through the vessels of the part, the phrase, *determination of blood*, is used to express the condition. In the act of blushing, there is determination of blood to the cheeks. When there is increased quantity of blood in a part, with diminished motion, the condition is called *congestion.* When there is increased quantity of blood in a part, with increased motion in some of its vessels, and diminished or obstructed motion in the others, the condition is *inflammation.*

Pain may be experienced in the dental pulp or periosteum, from any one of the above conditions. Any one of them will produce pressure on the sensitive nerve fibrils, the degree of pressure varying directly with the degree of departure from the normal condition, and the resistance of the surrounding parts. The pulp and the dental periosteum being increased in, or between bony textures, are far more painful, under the same degree of congestion or inflammation, than are similar tissues surrounded by muscle or membrane. *They have not room to swell.*

Cold applications to living tissue cause a contraction of its vessels. If the pain, resulting from pressure on the nerve fibrils, is caused simply by determination of blood, the contraction of the vessels forces out the excess of their contents, and the pressure is relieved, and, of course, the pain ceases. The same result follows the application, when congestion is present, though less promptly. But when inflammation is the predominant pathological condition, the motion of the blood through the vessels is obstructed—in some of them arrested, even; hence, the vessels are not able to force out their contents by contraction, and so contract on them, thus increasing, instead of diminishing, the pressure, and, therefore, aggravating instead of alleviating the pain.

With these views clearly before the mind, any one can appreciate the reasons why cold applications sometimes alleviate, and at other times aggravate the pain of toothache. And the feelings of the patient afford a guide to the treatment. While relieved by cold, the pathological state is not one of inflammation; and thus we are afforded a guide in diagnosis. Determination of blood, and congestion, may be overcome by a persevering use of cold; while, in inflammation, a treatment directly opposite is often indicated. Without trying to be technical, I hope I am understood.

